# Long-Acting Reversible Contraceptives: An Important Focus at the 2016 International Conference on Family Planning

**DOI:** 10.9745/GHSP-D-16-00241

**Published:** 2016-08-11

The fourth International Conference on Family Planning (ICFP) was held in Nusa Dua, Indonesia, on January 25–28, 2016. Themed “Global Commitments, Local Actions,” the conference stressed the importance of global and local partnerships to shape and influence the role and contributions of family planning in attaining health and development goals, in particular the new Sustainable Development Goals. Hosted by the Bill & Melinda Gates Institute for Population and Reproductive Health at Johns Hopkins Bloomberg School of Public Health and the National Population and Family Planning Board of Indonesia, the conference attracted more than 3,000 participants and featured more than 500 oral presentations, 400 posters, and 90 panels and plenaries.

We conceived this special issue of GHSP, comprising articles presented at ICFP, in collaboration with the Gates Institute to help disseminate important findings and lessons learned beyond conference participants to the larger health and development community. When reviewing the individual oral presentations accepted to the conference, we found at least 64 (13% of those accepted) that focused on long-acting reversible contraceptives (LARCs). It was clear to us that LARCs were an important focus of the conference and had unparalleled potential to reduce unmet need for family planning and improve contraceptive prevalence around the world.

This special issue includes 11 articles that focus specifically on programs that deliver LARCs and how they were implemented so that others can learn from their experience. The articles address a range of cross-cutting topics, from training, supervision, mentoring, and task sharing to quality of care (including removal of IUDs and implants when requested by the client or when the effective duration of the method ends), vouchers, postpartum family planning, postabortion family planning, and family planning service delivery in fragile environments. The countries featured in the articles include Bangladesh, Cambodia, Chad, the Democratic Republic of the Congo, Kenya, Pakistan, Senegal, Uganda, and Yemen.

This special issue is by no means an exhaustive review of the issues related to LARCs, but the articles provide important insight into the benefits and challenges of delivering LARC services, yielding important lessons that can be applied in other settings and that are also applicable to delivery of family planning services in general. *–Global Health: Science and Practice*

